# Obstructive sleep apnea, chronic obstructive pulmonary disease and hypertensive microvascular disease: a cross-sectional observational cohort study

**DOI:** 10.1038/s41598-022-17481-9

**Published:** 2022-08-03

**Authors:** Sky Chew, Deb Colville, Anastasia Hutchinson, Piers Canty, Lauren Hodgson, Judy Savige

**Affiliations:** 1 The University of Melbourne Department of Medicine, Northern Health and Melbourne Health, Royal Melbourne Hospital, Parkville, VIC 3050 Australia; 2 Northern Health, Epping, Australia; 3 The University of Melbourne Department of Ophthalmology Royal Victorian Eye and Ear Hospital, East Melbourne, Australia

**Keywords:** Biomarkers, Cardiology, Diseases, Pathogenesis, Risk factors

## Abstract

Hypertensive microvascular disease is associated with an increased risk of diastolic heart failure, vascular dementia and progressive renal impairment. This study examined whether individuals with obstructive sleep apnoea (OSA) had more retinal hypertensive microvascular disease than those with chronic obstructive pulmonary disease (COPD) and hospital controls. This was a single-centre, cross-sectional, observational study of participants recruited consecutively from a general respiratory clinic and a general medical clinic. OSA was diagnosed on overnight polysomnography study (apnoea:hypopnoea index ≥ 5), and controls with COPD had a forced expiratory volume/forced vital capacity (forced expiratory ratio) < 70%. Individuals with both OSA and COPD were excluded. Hospital controls had no COPD on respiratory function testing and no OSA on specialist physician questioning. Study participants completed a medical questionnaire, and underwent resting BP measurement, and retinal photography with a non-mydriatic camera. Images were deidentified and graded for microvascular retinopathy (Wong and Mitchell classification), and arteriole and venular calibre using a semiautomated method at a grading centre. Individuals with OSA (n = 79) demonstrated a trend to a higher mean arterial pressure than other hospital patients (n = 143) (89.2 ± 8.9 mmHg, p = 0.02), and more microvascular retinopathy (p < 0.001), and narrower retinal arterioles (134.2 ± 15.9 μm and 148.0 ± 16.2 μm respectively, p < 0.01). Microvascular retinopathy and arteriolar narrowing were still more common in OSA than hospital controls, after adjusting for age, BMI, mean arterial pressure, smoking history and dyslipidaemia (p < 0.01, p < 0.01, respectively). Individuals with OSA demonstrated a trend to a higher mean arterial pressure than those with COPD (n = 132, 93.2 ± 12.2 mmHg and 89.7 ± 12.8 mmHg respectively, p = 0.07), and more microvascular retinopathy (p = 0.0001) and narrower arterioles (134.2 ± 15.9 and 152.3 ± 16.8, p < 0.01). Individuals with OSA alone had more systemic microvascular disease than those with COPD alone or other hospital patients without OSA and COPD, despite being younger in age.

## Introduction

Obstructive sleep apnoea (OSA) affects 5–10% of middle-aged adults, and is even more common in the elderly^1–3^. Its prevalence is increasing with the obesity epidemic^4^. OSA is characterized by repeated episodes of partial or complete upper airways obstruction during sleep, due to relaxation of the tongue and airways muscles^5^. This leads to snoring, and a reduction (‘hypopneoa’) or blockage (‘apnoea’) of airflow. Apneoic episodes result in acute physiological stress including arterial desaturation, and surges of sympathetic activity^6^ with tachycardia and hypertension.

At least 50% of people with OSA have hypertension^7^, and hypertension worsens with more severe disease. OSA predisposes not only to nocturnal hypertension but also sustained daytime hypertension^8^. OSA increases cardiac risk and mortality^9–12^, independent of the traditional risk factors^13^. Type 2 diabetes is more common too because sleep-associated oxygen desaturation impairs glucose tolerance^14^ independent of obesity^15^. OSA also results directly in coronary microvascular dysfunction and subclinical coronary artery disease^16–19^ partly through oxidative stress^20^.

Chronic obstructive pulmonary disease (COPD) is another chronic respiratory disease but is characterized by inflammation in the airways and not fully reversible airflow obstruction. It affects 10% of the population over the age of 40^21^, most of whom have been smokers. Individuals with COPD have a two- to three- times increased risk of cardiac disease, but smoking alone does not explain this association, and shared genetic risk factors, inflammatory mechanisms, oxidative stress and neurohumoral responses have all been suggested. COPD also results in microvascular disease^22^. COPD overlaps with OSA and 10 -30% of individuals with COPD also have OSA^23^. Any study of the consequences of OSA on the retinal microvasculature must exclude individuals who also have COPD, but this is rarely undertaken.

Small vessel disease in the retina reflects systemic small vessel disease including the risk of cardiac events and stroke^24,25^. Small vessel changes are attributable not only to hypertension^26^ but also depend on the macrovascular risk factors, of age, gender, diabetes, smoking, family history and dyslipidemia. Features of small vessel disease include arteriovenous nicking, hemorrhage, exudates, and focal and generalized arteriolar narrowing (Keith-Wagener-Barker classification, modified by Wong and Mitchell^27^). However calibre is also affected by systemic inflammation, for example, with diabetes^28^ obesity^29^, and smoking^30^.

The advantages of retinal small vessel examination over other methods of vascular assessment are that it is accessible, fast, inexpensive and highly reproducible. The primary outcome of this study was thus to compare the occurrence of microvascular retinopathy in individuals with OSA alone, in hospital patients without OSA or COPD, or in individuals with COPD without OSA. The secondary outcome was to compare the effect of hypertension in OSA, COPD and other hospital patients on retinal microvascular calibre. Other studies have examined retinal microvascular disease in OSA^31–34^, but to date none has excluded individuals with both OSA and COPD; some used a self-administered questionnaire for diagnosis (‘sleep-disordered breathing’)^34^; one examined retinal photographs up to 3 years after the polysomnograms^31^; one diagnosed hypertension on history and did not take current BP into account^31^; and two did not consider diabetes or smoking when interpreting small vessel calibre^32,33^.

## Patients and methods

### Study design

This was a single centre, cross-sectional, observational study of consecutive individuals with OSA recruited from the respiratory or general medical clinic of a metropolitan teaching hospital over a 6 month period. Hospital patients without OSA or COPD and those with COPD were recruited from the same clinics.

Recruitment, data capture and retinal photographs were coordinated in a single episode during a clinic visit. Individuals diagnosed with OSA within the previous year but without COPD on respiratory function testing were invited to participate. Exclusion criteria were COPD on respiratory function testing (to enable us to determine microvascular disease in OSA alone) or ungradable retinal images.

Hospital controls and patients with COPD were used as controls because their vascular risk factors and respiratory function were known.

Participants were assisted to complete a structured questionnaire, and then underwent BP measurement, and retinal photography. Retinal images were deidentified, and examined for microvascular retinopathy and vessel calibre by trained graders. The presence and severity of retinal microvascular abnormalities and caliber measurements were compared between subjects with OSA, hospital controls without OSA or COPD, and subjects with COPD. There were no changes to the study design after its commencement and no interim analysis.

The study was approved by the Northern Health Human Research Ethics Committee according to the Principles of the Declaration of Helskinki, and all participants provided signed, informed consent.

### Participants

OSA was diagnosed on a polysomnography study (apnea: hypopnea index ≥ 5) in an accredited diagnostic sleep laboratory where all studies were interpreted by a specialist sleep physician and conducted within the previous year. Testing included electroencephalography, electrooculography, electromyography, electrocardiography, pulse oximetry, oral and nasal airflow, thoracic and abdominal motion using standardized criteria (Compumedics software, Melbourne). Apnea was defined as cessation of airflow lasting ≥ 10 s, and hypopnea as a decrease in tidal volume with a ≥ 4% reduction in oxyhaemoglobin saturation. The apnea: hypopnea index (AHI) was the average number of apneas plus hypopneas per hour of objectively- measured sleep. OSA was classified as mild (apnoea:hypopnea index at least 5 but < 15), moderate (at least 15 but < 30) or severe (at least 30). Other OSA features that were recorded included the Epworth sleepiness scale score (by questionnaire), hypoxia duration and REM-predominant sleep.

Hospital controls were patients who had no previous diagnosis of COPD, asthma or bronchiectasis and had normal respiratory function tests within the past year. Individuals with COPD were recruited consecutively from a general respiratory clinic and had been diagnosed with an FER (ratio of FEV_1_ to forced vital capacity) of < 70%. Respiratory function tests were performed using a computerized spirometer (Sensor Medics Legacy 29D, Yorba Linda, USA) by a trained technician according to a standard protocol^35,36^. Hospital controls and individuals with COPD were confirmed by a physician (JS) to have no clinical features of OSA, including excess daytime sleepiness, witnessed breathing interruptions, or awakenings due to gasping or choking^37^. These are features consistent with a negative score on the STOP-Bang questionnaire^38^.

Participants were assisted to complete a structured questionnaire for demographics (age, gender), and vascular risk factors (smoking, cigarette pack years, diabetes, hypertension, dyslipidemia), and laboratory test results (haemoglobin, lipids, estimated glomerular filtration rate) were obtained from their medical records. Hypertension was defined as a physician-made diagnosis, even if treated, and dyslipidemia with a cholesterol ≥ 5.0 mmol/l, HDL ≤ 2.0 mmol/l, or statin use. Blood pressure was recorded after 5 min sitting using a Hg sphygmomanometer, and mean arterial pressure and pulse pressure were calculated from the systolic and diastolic BP.

### Measurements

Retinal imaging was performed, and images graded for retinopathy. All participants underwent colour retinal photography using a non-mydriatic camera (KOWA 7, Japanor Canon CR5-45NM, Japan). Standard 45º images were taken of both eyes, with one view centred on the macula and another on the optic disc. All images were deidentiifed, and graded for microvascular retinopathy (Wong and Mitchell classification^27^ by an ophthalmologist and a trained observer. Microvascular retinopathy was classified as mild (arteriovenous nicking, focal arteriolar narrowing, silver-wiring or a decreased arteriovenous ratio), moderate (haemorrhage or exudates) or severe (papilloedema)^27^. In all cases the grade of the more severely-affected eye was used in the assessment.

Retinal images were sent to the Centre for Eye Research Australia for measurement of the retinal arteriole and venular calibre by trained graders using a standardized protocol and Knudtson’s revision of the Parr–Hubbard formula^39^. Briefly, all vessels passing through a zone 0.5–1 disc diameter from the optic disc margin were examined using a semi-automated computer imaging program (University of Wisconsin, WI), and measures based on the 6 largest vessels were combined into the Central Retinal Artery and Vein Equivalents (CRAE and CRVE). This method was highly reproducible.

### Statistical analysis

Demographic data were compared using Fisher’s exact test, chi square test or ANOVA. Possible determinants of calibre were examined using univariate analysis and then in multivariate linear regression models. Statistical analyses were performed using STATA version 11.2 software (STATACORP Inc, College Station Texas). A p value < 0.05 was considered significant and p < 0.10 a trend.

## Results

Seventy-nine subjects with OSA, 143 hospital controls, and 132 with COPD were studied (Table 1). Thirty-five further individuals with OSA (31%) were excluded because they also had COPD. Six (8%) and 13 (10%) subjects were excluded from the OSA and COPD cohorts respectively because their retinal images were ungradeable.

**Table 1:** Baseline demographic and clinical characteristics of subjects with OSA, hospital controls and subjects with COPD.

	OSA (n = 79)	Hospital controls (n = 143)	OSA versus hospital controls 95% CI, p value	COPD (n = 132)	OSA versus COPD, 95% CI, p value
**Clinical characteristics**					
Age (mean, SD, years)	**62.5, 12**	**69.3, 7.7**	**4.2–9.41, < 0.01**	**69.9, 9.7**	**4.2–10.1, < 0.01**
Gender (male)	44 (56%)	75 (52%)	1.14, 0.63–2.06, 0.64	66 (50%)	1.26, 0.7–2.3, 0.43
BMI (mean, SD, kg/m^2^)	**35.9, 8.5**	**26.5, 6**	**− 11.30 to − 7.41, < 0.01**	**27.1, 6.6**	**− 10.8 to − 6.6, 0.01**
Smoking history	**42 (53%)**	**77 (54%)**	**1.03, 0.57–1.87, 0.92**	**126 (96%)**	**0.06, 0.02–0.2, 0.01**
Pack years (mean, SD)	**31.6, 19.5**	**33, 25.4**	**− 7.97 to 10.73, 0.77**	**46.3, 26.4**	**5.5–23.8, 0.01**
Hypertension diagnosis	56 (71%)	75 (52%)	2.21, 1.19–4.17, 0.01	81 (61%)	1.53, 0.81–2.94, 0.16
Mean arterial pressure (mean, SD, mmHg)	**93.2, 12.2**	**89.2, 8.9**	**0.65–7.2, 0.02**	**89.7, 12.8**	**− 0.32 to 7.28, 0.07**
Mean pulse pressure (mean, SD, mmHg)	**54.5, 13.5**	**55.2, 13.4**	**− 89.82 to 69.42, p = 0.74**	**60.1, 16.9**	**− 10.36 to − 0.84, 0.02**
Diabetes	25 (32%)	34 (24%)	1.48, 0.77–2.85, 0.21	32 (24%)	1.45, 0.74–2.81, 0.24
Dyslipidemia	**43 (54%)**	**43 (30%)**	**2.78, 1.51–5.10, < 0.01**	**55 (42%)**	**1.67 (0.92–3.05), 0.07**
**Retinal features**					
No retinopathy	2 (3%)	72 (50%)	0.57, 0.19–1.62, < 0.01	27 (20%)	1.26, 0.73–2.17, 0.41
Any retinopathy	**77 (97%)**	**72 (50%)**	**< 0.001**	**105 (80%)**	**< 0.001**
Mild	**60 (76%)**	**54 (38%)**	**< 0.001**	**67 (51%)**	**0.003**
Moderate	17 (21%)	17 (12%)	0.08	38 (29%)	0.26
Severe	0 (0%)	0 (0%)		0 (0%)	
Arteriole caliber (mean, SD, µm)	**134.2, 15.9**	**148, 16.2**	**− 31.2 to − 12.3, < 0.01**	**152.3, 16.8**	**− 17.0 to − 5.9, < 0.01**
Venular caliber (mean, SD, µm)	**197.3, 24.0**	**213.9, 26.2**	**− 44.9 to − 19.1, 0.23**	**224.8, 28.1**	**− 24.7 to − 7.0, < 0.01**

Significant values are in bold.

### Subjects with OSA

Fifty-six (71%) subjects with OSA had hypertension, with an overall mean arterial pressure of 93.2 ± 12.2 mmHg (Table 1). Their mean BMI was 35.9 ± 8.5 kg/m^2^ and 25 (32%) had diabetes. Twenty (25%) had mild, 22 (28%) had moderate and 37 (47%) had severe OSA. Their mean Epworth Sleepiness Scale was 10.4 ± 5.3 (2–24), 16 (20%) had an Epworth Sleepiness Scale ≥ 15, and their mean hypoxia duration was 8.7 ± 17.9 s. Fifty-three (67%) were prescribed CPAP but it was unclear how many adhered to treatment adequately.

**Figure 1:**
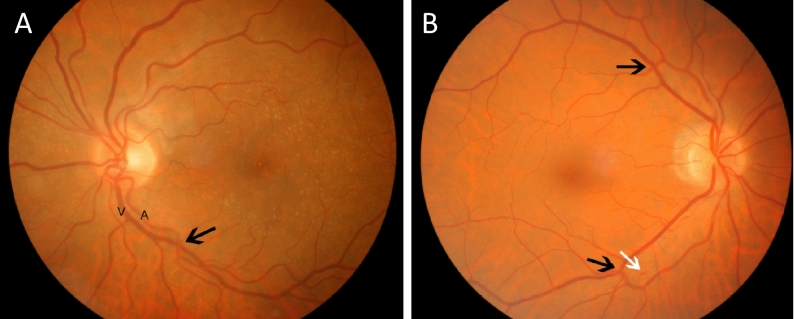
Retinal imaging demonstrating. (**A**) Mild microvascular retinopathy with arteriole (A) and vein and arteriovenous nicking (black arrow); and (**B**) moderate microvascular retinopathy with further examples of microvascular nicking (black arrows) and likely haemorrhage (white arrow).

**Figure 2:**
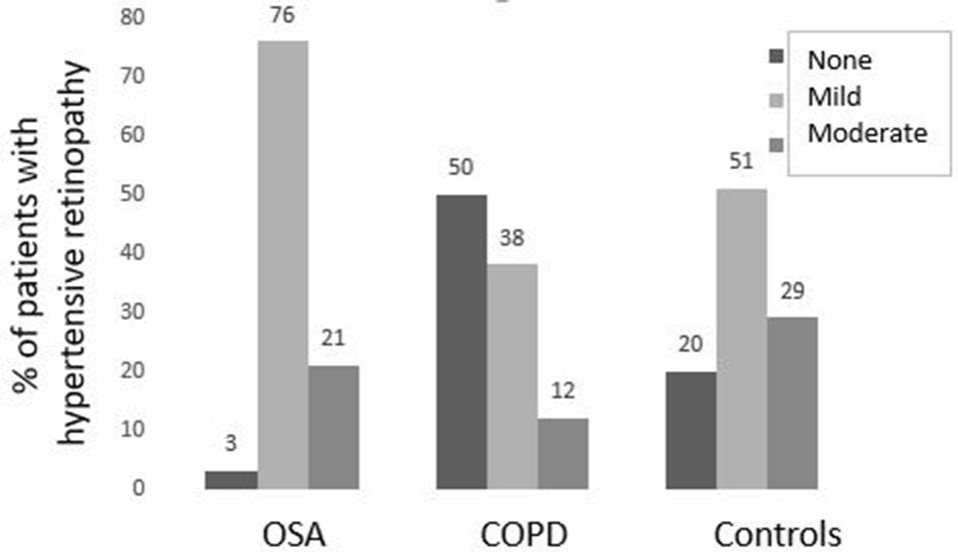
% of individuals with no, mild or moderate microvascular retinopathy in OSA, Controls and COPD.

Almost all subjects with OSA (97%) had a retinal microvascular retinopathy with mild (60, 76%) or moderate (17, 21%) features (Table 1, Figs. 1, 2). Moderate microvascular retinopathy was not associated with age, smoking history, diabetes, hypertension, dyslipidema or BMI (p all > 0.05). Moderate retinopathy was also not associated with OSA features (severity, p = 1.00), increased Epworth Sleepiness Scale score (p = 0.56), longer hypoxia duration (p = 0.49) or more REM-predominant sleep (p = 1.00).

Subjects with OSA had a mean retinal arterial calibre of 134.2 ± 15.9 µm and mean retinal venular calibre of 197.3 ± 24.0 µm. Those with OSA and a moderate microvascular retinopathy had narrower arterioles and venules than those without a retinopathy (136.0 ± 14.6 µm and 165.0 ± 9.7 µm respectively, 95% CI 9.0–54.3 µm, p < 0.01; and 198.8 ± 13.5 µm and 250.0 ± 52.2 µm, 95% CI 18.3–86.3, p < 0.01) (Table 2).

**Table 2:** Correlation of Microvascular retinopathy with OSA features and retinal arteriolar and venular calibre.

Feature	Retinopathy			p value
OSA (n = 79)	None (n = 2)	Mild (n = 61)	Moderate (n = 13)	
Mild (n = 20)	**0**	**14**	**3**	0.97
Moderate (n = 22)	**1**	**18**	**3**	
Severe (n = 37)	**1**	**29**	**7**	
CRAE (mean, SD)	**165.0, 9.7**	**132.7, 16.4**	**136.0, 14.6**	**< 0.01**
CRVE (mean, SD)	**250.0, 52.2**	**197.7, 25.2**	**198.8, 13.5**	**< 0.01**

More severe grades of OSA based on the apnoea: hypoponoea index were not associated with more severe microvascular retinopathy. However arteriolar and venular calibre were narrower with mild and moderate retinopathy compared with none.

*CRAE* central retinal artery equivalent, *CRVE* central retinal vein equivalent.

Significant values are in bold.

When subjects with OSA alone were examined according to quartiles of their retinal arteriole calibre, there were no differences between the quartiles in OSA severity measured by apnoea:hypoponea index (p = 1.00), Epworth Sleepiness Scale (p = 0.38), hypoxia duration (p = 0.24), or REM-predominant sleep (p = 0.50). These observations suggest that the retinal arteriole narrowing did not worsen directly with any of the commonly-used measures of OSA severity.

When subjects with OSA alone were examined according to quartiles of their retinal venular calibre, there were again no differences between the quartiles in OSA severity measured by apnoea; hypopnoea index (p = 1.00), Epworth Sleepiness Scale (p = 0.52), hypoxia duration (p = 0.89), or REM predominant sleep (p = 0.07). Again these observations suggest that the retinal venular narrowing did not worsen directly with any of the commonly-used measures of OSA severity.

### Subjects with OSA compared with hospital controls

Subjects with OSA were younger (p < 0.01), had a higher BMI (p < 0.01), more dyslipidemia (p = 0.01), more hypertension (p = 0.01) and a higher mean arterial pressure (p = 0.02) than hospital controls (Table 1, Fig. 2). Their likelihood of smoking, diabetes or a higher pulse pressure were not different.

Subjects with OSA had more microvascular disease than other hospital patients (p < 0.001). They had more mild retinopathy (p < 0.001), and there was a trend for more moderate retinopathy (p = 0.08). Microvascular retinopathy was still more common in subjects with OSA than hospital controls after adjusting for age, BMI, hypertension (p = 0.04) and dyslipidemia (p = 0.03).

Subjects with OSA had a smaller mean arteriole calibre than other hospital patients (134.2 ± 15.9 µm and 148.0 ± 16.2 µm, respectively, mean difference − 31.2 to − 12.3 µm, p < 0.01), but venular calibre was not different (197.3 ± 24.0 µm and 213.9 ± 26.2 µm, 95% CI − 44.9 to -19.1 µm, p = 0.23).

When features in subjects with OSA plus hospital controls were compared by quartile of arteriolar calibre, a smaller calibre was associated with hypertension (p = 0.04), lower BMI (p = 0.02), non-smoking status (0.06), and smaller venular calibre (p < 0.01) (Table 3). For the corresponding quartiles in venular calibre, smaller vessels correlated with increasing age (p = 0.01), fewer cigarette pack years (p = 0.07) and smaller arterioles (p < 0.01) (Table 4).

**Table 3:** Clinical features and retinal arteriole calibre in OSA plus hospital controls by quartile.

Retinal arteriole calibre					
Clinical features in OSA and controls	90–129 µm (n = 48)	130–140 µm (n = 49)	141–155 µm (n = 49)	156–190 µm (n = 48)	OR, 95%CI, p value
Age (mean, SD, years)	66.9, 9.4	62.7, 11.1	69.1, 8.4	66.7, 8.6	0.93
Gender (male)	31 (65%)	26 (53%)	21 (43%)	22 (46%)	0.10
BMI (mean, SD, kg/m^2^)	31.1, 6.6	32.5, 9.4	28.2, 6.8	27.5, 7.6	**0.02**
Smoking history	21 (44%)	26 (53%)	28 (57%)	31 (65%)	**0.06**
Pack years (mean, SD)	32.5, 21.5	34.2, 17.8	27.9, 21.3	35.8, 32	0.7
Hypertension history	33 (69%)	26 (53%)	29 (59%)	22 (46%)	**0.04**
Mean arterial pressure (mean, SD, mmHg)	92.7, 13.2	91.8, 11.4	90.7, 10	89, 8	0.17
Dyslipidemia	16 (35%)	23 (47%)	20 (41%)	15 (31%)	1.00
Diabetes	11 (23%)	16 (33%)	12 (25%)	13 (27%)	0.80
Retinal venular calibre (mean, SD, µm)	189.6, 18.4	202, 20	211.2, 25.1	229, 23.8	**< 0.01**
**OSA patients only**					
OSA (n = 79)	n = 34	n = 29	n = 10	n = 6	< 0.001
Mild (n = 20)	7 (9%)	8 (10%)	3 (4%)	2 (3%)	0.12
Moderate (n = 22)	9 (11%)	8 (10%)	4 (5%)	1 (1%)	0.04
Severe (n = 37)	18 (23%)	13 (16%)	3 (4%)	3 (4%)	< 0.001
Mean pulse pressure (mean, SD, mmHg)	58.4, 13	56.1, 13	52.5, 17	51.0, 13	0.60

Smaller retinal arteriole calibre was associated with a higher BMI (p = 0.02), a hypertension diagnosis (p = 0.04) and a smaller venular calibre (p < 0.01), and there was a trend with less smoking history (p = 0.06).

In the patients with OSA, all OSA was associated with a smaller arteriole calibre (p < 0.001). Moderate and severe OSA were associated with a smaller arteriole calibre (p = 0.04, p < 0.001 respectively). Retinal arteriole calibre was not associated with mean pulse pressure (p = 0.60).

Significant values are in bold.

**Table 4:** Clinical features and retinal venular calibre in OSA plus hospital controls by quartile.

Retinal venular calibre					
Clinical features in OSA and controls	156–189 µm (n = 48)	190–205 µm (n = 49)	206–223 µm (n = 49)	224–291 µm (n = 48)	p value
Age (mean, SD, years)	68.8, 9.3	67, 8.7	65.7, 10.8	64, 9.2	**0.01**
Gender (male)	25 (52%)	26 (53%)	21 (43%)	28 (58%)	0.68
BMI (mean, SD, kg/m^2^)	29.5, 8.2	30.6, 7.4	28.6, 6.4	30.5, 9.5	0.58
Smoking history	21 (44%)	25 (51%)	29 (59%)	28 (58%)	0.22
Pack years (mean, SD)	24.6, 19.6	34.5, 19.5	30.7, 21	39, 31.5	**0.07**
Hypertension	32 (67%)	29 (59%)	24 (49%)	25 (52%)	0.21
Mean arterial pressure (mean, SD, mmHg)	88.2, 11.8	94.7, 10.3	89.8, 11.3	91.3, 9.4	0.24
Dyslipidemia	19 (40%)	22 (45%)	14 (29%)	19 (40%)	1.00
Diabetes	15 (31%)	12 (25%)	8 (16%)	16 (33%)	1.00
Retinal arteriole calibre (mean, SD, µm)	130.4, 15.2	136.4, 13	147.5, 14.1	157.1, 15.1	**< 0.01**
**OSA patients only**					
OSA (n = 79)	n = 31	n = 21	n = 12	n = 6	< 0.001
Mild (n = 20)	6	9	2	0	0.02
Moderate (n = 22)	8	7	5	1	0.11
Severe (n = 37)	17	5	5	5	< 0.001
Mean pulse pressure (mean, SD, mmHg)	55.8, 15.5	57.5, 14	55.0, 7.0	54.6, 14.0	0.98

Smaller retinal venular calibre was associated with an older age (p = 0.01), a smaller arteriole calibre (p < 0.01), and there was a trend with fewer pack years (p = 0.07).

In patients with OSA only, all OSA was associated with a smaller venular calibre (p < 0.001). Mild and severe OSA were associated with a smaller venular calibre (p = 0.02, p < 0.001 respectively). Retinal venular calibre was not associated with mean pulse pressure (p = 0.98).

Significant values are in bold.

Microvascular retinopathy and narrowed arterioles and venules were still more common in subjects with OSA than other hospital patients after adjusting for possible confounders (p < 0.01, p < 0.01 and p < 0.01) (Table 5).

**Table 5:** Determinants of microvascular retinopathy, and vessel caliber in subjects with OSA, hospital controls or with COPD in a multivariate analysis.

	(a) OSA and hospital controls			(b) OSA and COPD		
	OR	95% CI	p value	OR	95% CI	p value
**Microvascular retinopathy**	27.17	5.62 to 131.4	< 0.01	6.67	1.27 to 34.82	0.02
Age	1.03	0.98 to 1.07	0.24	1.01	0.97 to 1.06	0.40
BMI	1.05	0.99 to 1.11	0.11	1.02	0.95 to 1.09	0.59
Smoking history				0.37	0.04 to 3.17	0.36
Hypertension	2.07	1.03 to 4.17	0.04	1.17	0.49 to 2.77	0.73
Dyslipidemia	2.29	1.09 to 4.81	0.03	0.60	0.26 to 1.39	0.23
**Retinal arteriole caliber**	− 13.19	− 19.25 to − 7.13	< 0.01	− 16.67	− 23.2 to − 10.12	< 0.01
Age	− 0.17	− 0.45 to 0.11	0.24	− 0.39	− 0.65 to − 0.15	< 0.01
BMI	− 0.09	− 0.46 to 0.27	0.62	− 0.31	− 0.67 to 0.06	0.10
Smoking history				3.51	− 3.36 to 10.41	0.32
Hypertension	− 1.6	− 6.81 to 3.47	0.52	− 2.09	− 7.55 to 3.36	0.45
Dyslipidemia	0.06	− 4.90 to 5.02	0.98	− 0.28	− 5.32 to 4.76	0.91
**Retinal venular caliber**	− 19.94	− 29.04 to − 10.87	< 0.01	− 27.13	− 37.57 to − 16.68	< 0.01
Age	− 0.64	− 1.06 to − 0.23	< 0.01	− 0.80	− 1.20 to − 0.40	< 0.01
BMI	0.33	− 0.22 to 0.89	0.24	− 0.18	− 0.77 to 0.40	0.53
Smoking history				8.27	− 2.68 to 19.27	0.14
Hypertension	− 1.95	− 9.65 to 5.76	0.62	− 0.19	− 8.88 to 8.51	0.97
Dyslipidemia	− 0.92	− 8.36 to 6.51	0.81	2.38	− 5.65 to 10.42	0.56

This study included a.individuals with OSA or hospital controls or b. individuals with OSA or COPD; and examined for microvascular retinopathy, retinal arteriole calibre or retinal venular calibre taking into account the same variables for each that were demonstrated in Tables 3 and 4 (age, BMI, smoking history, hypertension diagnosis and dyslipidemia).

### Subjects with OSA compared with COPD

Subjects with OSA were younger, had a higher BMI, and less cigarette exposure than those with COPD (p all < 0.001) (Table 1, Fig. 2). They were not more likely to have a diagnosis of hypertension (p = 0.16) or diabetes (p = 0.24), but there were trends to a higher mean arterial pressure (p = 0.07), more dyslipidemia (p = 0.07) but a lower pulse pressure (p = 0.02) than those with COPD.

Subjects with OSA had more microvascular retinopathy (p < 0.001) than those with COPD alone (Table 1). Microvascular retinopathy was still more common after adjusting for possible confounders (OR 6.67, 95% CI 1.27–34.82, p = 0.02) (Table 5).

Subjects with OSA also had narrower arterioles (134.3 ± 16.7 µm and 152.3 ± 16.8 µm, respectively, mean difference − 18.0 µm, − 12.9 to -23.1 µm, p < 0.01) and venules than those with COPD (199.5 ± 25.2 µm and 224.8 ± 28.1 µm, mean difference − 25.3, − 17.1 to − 33.5 µm, p < 0.01). Retinal arteriole and venular calibre were still less after adjusting for potential confounders (Table 5).

## Discussion

This study found more systemic small vessel disease in individuals with OSA than in other hospital patients. It also found more small vessel disease in OSA after patients with COPD were excluded than in those with COPD alone. Nearly all subjects with OSA in this study had a microvascular retinopathy and narrowed arterioles despite being younger than the other cohorts. The increase in retinal small vessel disease in OSA is consistent with the increased risk of systemic microvascular disease associated with diastolic heart failure, vascular dementia and progressive renal failure^40–42^.

Individuals with OSA had more microvascular retinopathy and narrower arterioles than hospital controls and the COPD cohort. Poorly-controlled hypertension may be a contributor to the small vessel disease seen in OSA. Diagnosed hypertension was more common in OSA than hospital controls. The mean arterial pressure was higher in OSA than the hospital controls and those with COPD. However pulse pressure which corresponds to arterial stiffness^43^ was not higher in OSA than in the other cohorts.

These results suggest a disparity between diagnosed hypertension, clinic blood pressure readings and retinal microvascular changes. Possible explanations include that the blood pressure was measured only once in this study during a clinic visit and that patients were more likely to take their antihypertensive medication on the day of a medical appointment. Secondly, previous reports of an association between OSA and hypertension did not exclude individuals with COPD which will have distorted the results. Thirdly, smoking in COPD increases both venular and arteriole calibre and complicates the measurement of vessel calibre. Finally and importantly, hypertension control in OSA is difficult to assess since hypertension is mainly nocturnal.

Repetitive apnoeas in OSA at night increase the sympathetic drive and trigger fluctuations in blood pressure and heart rate^44^. They also suppress nitric oxide production^45^. The mechanism of vascular disease may be through the release of free radicals, and reduced vasodilation. Endothelial dysfunction is a key event that precedes atherosclerosis and represents a pathogenic link with cardiovascular disease^46^. In addition the hypertension in OSA is now correlated more directly with the damage from intermittent hypoxemia and ischemic reperfusion injury^47^ rather than the sustained hypoxemia seen in COPD.

This study also did not demonstrate worse microvascular retinopathy or a consistently narrower calibre with more ‘severe’ OSA as assessed by an apnoea:hypopnea index > 30. Possible explanations include that the cohort included many individuals with milder OSA, the sleep studies were performed prior to treatment, and that CPAP was commonly prescribed^48^. In diseases other than OSA, improved blood pressure control reverses small vessel abnormalities^49^, and in OSA, CPAP treatment reverses the microvascular dysfunction^50,51^.

OSA coexists with COPD in up to 30% of individuals but this study minimised the risk of overlap by specifically questioning all participants for features of OSA and testing them for COPD. This study is, we believe, the first to examine microvascular disease in OSA independent of COPD. Arterioles and venules are usually larger in COPD^52^ because of vascular remodelling and intimal and medial thickening from the accumulation of inflammatory cells and fibroblasts^53^. The increased calibre demonstrated here in COPD compared with hospital controls or those with OSA reflects this exaggeration.

The strengths of this study were its high recruitment rate; the completeness of the data; the near contemporaneous nature of the sleep studies and retinal imaging; the robustness of the retinal microvascular assessments; and the use of multiple measures of hypertension. The control cohorts of hospital patients and subjects with COPD were chosen because their medical comorbidities were well-characterised. Some other studies have not even considered the diagnosis of hypertension, whereas we included a previous diagnosis of hypertension, and even treated hypertension since treatment is often inadequate. The measurement of hypertension in OSA is further complicated by its nocturnal nature. We also examined the effect of mean arterial pressure, which weights systolic and diastolic BP, and considered the effect of the blood pressure in the different cohorts.

The study’s major limitations were its cross-sectional and single centre nature, and the exclusion of OSA by clinical questioning rather than with sleep studies. It was difficult to evaluate an effect of CPAP since few patients appeared strictly adherent to treatment. However other studies have demonstrated a beneficial effect of 3–12 months treatment with CPAP on small vessel disease^54,55^.

The number of participants recruited was typical of studies examining retinal microvascular calibre in OSA. The proportion of patients with OSA excluded clinically with COPD or with ungradeable retinal images approximated previously reported frequencies. Diabetes is a common comorbidity in OSA occurring in 24–86% of many cohorts^56^, and OSA exacerbates diabetic retinopathy where this is present^57^. Our analysis corrected for diabetes but did not exclude this group so that the cohort was representative of all patients found in a respiratory clinic. Indeed the proportion of individuals with diabetes was not different in the cohorts with OSA (32%), COPD (24%) or the controls (24%).

This study suggests that subjects with OSA have an increased risk of small vessel disease that is greater than is found in COPD or in other hospital patients. These comparisons indicate where physician time and hospital resources should be directed in terms of minimising the risks of systemic small vessel disease.

## Data Availability

All deidentified data used and analysed in the current study is available from the corresponding author on reasonable request.
